# Comparative Genomics of the Mucoid and Nonmucoid Strains of Streptococcus pyogenes, Isolated from the Same Patient with Streptococcal Meningitis

**DOI:** 10.1128/genomeA.00221-15

**Published:** 2015-04-16

**Authors:** Haruno Yoshida, Yasuhito Ishigaki, Asako Takizawa, Kunihiko Moro, Yuki Kishi, Takashi Takahashi, Hidenori Matsui

**Affiliations:** a Department of Infection Control and Immunology and Graduate School of Infection Control Sciences, Kitasato Institute for Life Sciences, Kitasato University, Minato-ku, Tokyo, Japan; b Division of Molecular and Cell Biology, Medical Research Institute, Kanazawa Medical University, Uchinada-machi, Kahoku-gun, Ishikawa, Japan; c Division of Basic Research, Biomedical Laboratory, Kitasato Institute Hospital, Kitasato University, Tokyo, Japan; d Hikone Municipal Hospital, Hassaka-cho, Hikone-shi, Shiga, Japan

## Abstract

Mucoid (MTB313) and nonmucoid (MTB314) strains of group A streptococcus *emm* type 1 were simultaneously isolated from a single patient suffering from streptococcal meningitis. Whole-genome sequencing revealed that MTB313 carried a nucleotide substitution within *rocA*, which generated an amber termination codon.

## GENOME ANNOUNCEMENT

We identified mucoid (MTB313) and nonmucoid (MTB314) strains of group A streptococcus (GAS) *emm* type 1 at the same time on the same blood agar plate with application of a cerebrospinal fluid specimen of a 70-year-old Japanese woman suffering from streptococcal meningitis. We performed comparative genomics based on whole-genome sequencing of both GAS strains to comprehensively identify the naturally occurring nucleotide substitutions.

Genomic DNA samples of MTB313 and MTB314 were subjected to shotgun pyrosequencing by using a 454 GS Junior titanium system (454 Life Sciences, Branford, CT). All operations were carried out according to the protocols provided by the manufacturer. A putative annotation of the genome sequence was made using the Microbial Genome Annotation Pipeline (MiGAP; an auto annotation pipeline of the DDBJ, http://www.migap.org/). The sequences from MTB313 and MTB314 were analyzed for the detection of local and structural variations compared to those of MGAS5005 (a virulent type strain of *emm*1 GAS) (1) by using the GS Reference Mapper application (analysis software included with the 454 system).

The genome sizes of MTB313 and MTB314 (1,745,332 bp and 1,744,827 bp, respectively) were smaller than the previously completed genomes (1.84 Mbp in average size) of four other *emm*1 GAS strains (SF370 [2], MGAS5005 [1], A20 [3], and 476 [4], GenBank accession numbers AE004092, CP000017, CP003901, and AP012491, respectively) because of the lack of phage genomes. The comparative genomic analysis between MGAS5005 and MTB313 or MTB314 revealed that MTB313 carried a nucleotide substitution within *rocA* (G464A), which generated an amber chain-termination codon (UAG) at the 155th amino acid position (accession no. AB737848). In contrast, the same nucleotide sequences of *rocA* were detected in both MTB314 (accession no. AB737849) and MGAS5005 (gene ID 3571595).

*covR*-*covS* (control of virulence; also called *csrR*-*csrS*, capsule synthesis regulator), a two-component regulatory system (*covR* is the transcriptional regulator and *covS* is the sensor kinase), influences the expression of chromosomal genes of GAS (5–7). *covR* phosphorylated by *covS* is believed to negatively regulate the expression of several virulence genes of GAS, including *hasA* (hyaluronic acid capsule) (7). *rocA* has been reported to be a positive regulator of *covR* (8). Recently, Lynskey et al. (9) demonstrated that the *rocA* of serotype M18 GAS exhibited structural homology to the catalytic domain of the *Escherichia coli* osmoregulator *envZ*. Although *rocA* was shown to positively enhance *covR* transcription, quantitative proteomics revealed that *rocA* was a metabolic regulator with activity beyond the *covR*-*covS* regulon. A naturally occurring truncation of *rocA* contributed to the hyperencapsulation phenotype, led to prolonged nasopharyngeal carriage of GAS in mice, and promoted bacterial airborne transmission (9). These results suggest that MTB313 is a highly encapsulated phenotype associated with *hasA* expression through the suppression of *covR* expression by the depression of *rocA*.

### Nucleotide sequence accession numbers. 

The complete whole-genome sequences of MTB313 and MTB314 were registered to the DDBJ/ENA/GenBank database under the genome project data accession numbers AP014572 and AP014585, respectively.
